# treA Codifies for a Trehalase with Involvement in *Xanthomonas citri* subsp. *citri* Pathogenicity

**DOI:** 10.1371/journal.pone.0162886

**Published:** 2016-09-09

**Authors:** André Vessoni Alexandrino, Leandro Seiji Goto, Maria Teresa Marques Novo-Mansur

**Affiliations:** Laboratório de Bioquímica e Biologia Molecular Aplicada – LBBMA, Departamento de Genética e Evolução, Universidade Federal de São Carlos, São Carlos, SP, Brazil; Fujian Agriculture and Forestry University, CHINA

## Abstract

Citrus canker, caused by the bacterium *Xanthomonas citri* subsp. *citri* (Xcc), is a severe disease of citrus. Xcc presents broad spectrum of citrus hosts including economically important species whereas *X*. *fuscans* subsp. *aurantifolii–*type C (XauC) causes a milder disease and only infects *Citrus aurantifolia*. Trehalase catalyzes hydrolysis of the disaccharide trehalose, a sugar that has been reported to be related to Xcc pathogenicity. We expressed the recombinant gene product and assessed Xcc trehalase structural and kinetics data. The recombinant protein presented 42.7% of secondary structures in α-helix and 13% in β-sheets, no quaternary structure in solution, and Michaelis-Menten constant (K_M_) of 0.077 mM and V_max_ 55.308 μMol glucose.min^-1^.mg protein^-1^ for trehalose. A Xcc mutant strain (XccΔtreA) was produced by gene deletion from Xcc genome. Enzymatic activity of trehalase was determined in Xcc, XauC and XccΔtreA cellular lysates, showing the highest values for XauC in *in vitro* infective condition and no activity for XccΔtreA. Finally, leaves of *Citrus aurantifolia* infected with XccΔtreA showed much more drenching and necrosis than those infected by wild type Xcc. We concluded that trehalase contributes to alleviate bacterial virulence and that inability for trehalose hydrolysis may promote higher Xcc infectivity.

## Introduction

Citrus canker is one of the most important citrus diseases and causes reduced productivity and quality of citrus fruits, aggravated by the lack of effective measures to control and cure the disease [1]. *Xanthomonas citri* subsp. *citri* (Xcc) attack all known species of citrus and their hybrids, causing the most severe form of the disease (canker A). *Xanthomonas fuscans* subsp. *aurantifolii* type C (XauC) causes the less severe canker C that attacks a single host, *Citrus aurantifolia* [2, 3]. Canker A injuries the aerial parts of the plants, especially the leaves and the surface of fruits, causing disruption of the plant epidermis [1, 4]. The resulting eruptions can be surrounded or not by a yellow halo, associated with the decrease of chlorophylls a and b, carotene and xanthophyll [5]. Man is an important disease spreader through contaminated equipment handling, but natural events like rain also acts as a spreader, since water droplets collide with lesions carrying away the pathogen [1, 6].

Studies of citrus canker can rely on available Xcc genomic data [7–9] in order to better understand the mechanisms of bacterial infection. Functional studies of Xcc proteins involved with bacterial pathogenicity may be important to gather information in order to prevent the citrus canker.

Trehalases (EC 3.2.1.28) are enzymes that catalyze the hydrolysis of trehalose, a non-reducing disaccharide (α-D-glucopyranosyl-1,1-α-D-glucopyranoside), resulting in two glucose monomers [10]. Trehalose is a sugar widely distributed, being found in bacteria, fungi, animals (except mammals) and plants. This disaccharide plays different biological functions, from energy reserve, protein and membrane protection against various environmental stresses (drying, freezing, osmotic stress and starvation) to signaling and roles on growth pathways [10–12]. Trehalase (TreA) seems to be related to the response to environmental perturbations [13], which could eventually be related to Xcc response to the stress imposed by the host during the colonization.

Xcc genome (strain 306) has one ORF annotated as periplasmic trehalase (XAC0604, re-annotated as XAC_RS03145). To study this enzyme, we have expressed the recombinant gene product in *E*. *coli* and characterized secondary and quaternary structures and enzyme activity of the recombinant protein, as well as trehalase activity in Xcc and XauC lysates. A Xcc deletion mutant for trehalase (XccΔtreA) was also generated to study the relationship between this ORF and Xcc pathogenicity.

## Materials and Methods

### Bacterial strains and culture mediums

Strains Xcc 306 [7] and XauC 10535 (IBSBF338) [3] were provided by Fundecitrus (www.fundecitrus.com.br) and stored at -80°C in Luria Bertani (LB, Difco) supplemented with 10% glycerol (v/v). LB medium was also used as non-*hrp*-inducing medium. XAM-M, a *hrp*-inducing medium (Artier et al., unpublished results) composed by (NH_4_)_2_SO_4_ 7.57 mM; KH_2_PO_4_ 33.06 mM; K_2_HPO_4_ 60.28 mM; sodium citrate (C_6_H_5_Na_3_O_7_.2H_2_O) 1.7 mM; MgSO_4_ 1 mM; casamino acids 0.03%; fructose 10 mM; sucrose 10 mM; Bovine Serum Albumin (BSA) 1 mg.mL^-1^; adjusted to pH 5.4, was used to obtain bacterial cultures for native trehalase activity measurements. *E*. *coli* strains DH5α and BL21(DE3) were grown on Luria Bertani agar and broth.

### General methods

Xcc genomic DNA was purified with Wizard Genomic DNA Purification Kit (Promega). DNA polymerase (Phusion High Fidelity DNA polymerase), restriction enzymes, cloning vectors and other DNA extraction kits were purchased from Fermentas-Thermo Scientific. pNPTS138 vector was gently supplied by Prof. Dr. Ferreira H. (UNESP, Rio Claro-SP, Brazil). Custom oligonucleotides were provided by IDT. Ampicillin and kanamycin were purchased from Sigma. Molecular biology techniques followed standard methods [14] or are detailed.

### Cloning and construction of expression system

The pair of oligonucleotides for the amplification of the coding region of the Xcc TreA was designed based on the sequence deposited in GenBank (http://www.ncbi.nlm.nih.gov/genbank/), access code XAC0604/AAM35493.1. Bases were added to the extremity of each oligonucleotide in order to include an *Nde*I restriction site (underlined) at 5' end of the PCR product (5’ TAACATATGGCGCCGCTGGACGCTCCGGTC) and an *Xho*I site at 3' end (5’ TATCTCGAGTCAGCGCGCGGCCGCCTC), after the predicted stop codon. *In silico* analysis of the ORF XAC0604 using SignalP 4.0 [15] predicted an *N-*terminal signal peptide, which was not included for oligonucleotides design.

PCR amplification was carried out in a thermocycler C1000 Touch (Bio-Rad) using 500 ng of Xcc genomic DNA as template and 100 pmol of each oligonucleotide. The thermocycler program started with an initial denaturation at 97°C for 10 min, followed by 35 cycles of 94°C for 30 s, 61.5°C for 30 s, 72°C for 2 min and the final elongation step of 10 min at 72°C.

The purified amplification product was cloned into pJET 1.2 (Fermentas) and transformed into *E*. *coli* DH5α for propagation. The plasmid insert was fully confirmed by sequencing [16] in a 3130 Genetic Analyzer (Applyed Biosystems). The confirmed clone was digested with *Nde*I and *Xho*I and the excised insert was subcloned into pET28a (Novagen) previously digested with the same restriction enzymes. The built plasmid (pET28a_TreA) allows IPTG-induced expression of TreA fused to an *N-*terminal His-tag.

### Recombinant expression and protein purification

The pET28a_TreA plasmid was transformed into *E*. *coli* BL21 (DE3) (Novagen) and expression was carried out in 250 mL of LB broth added of kanamycin 40 μg.mL^-1^ in an orbital shaker at 250 rpm and 18°C. IPTG (0.1 mM) was added to culture in mid-log growth phase and after 16 h the cells were collected by centrifugation and resuspended in 50 mL of buffer I (25 mM Tris-HCl pH 8.0, 100 mM NaCl). The cells were lysed by ultrasound pulses under ice bath.

For IMAC purification (S1 Fig), the soluble fraction of the lysate was separated from cell debris by centrifugation (at 12,000 x *g* for 10 min at 4°C) and loaded onto a 5 mL Ni-NTA column (Novagen) pre-equilibrated with the buffer I. Then, the column was washed with 30 mL of 5 mM imidazole in the same buffer. For elution of the recombinant TreA were applied 30 mL of 100 mM imidazole in buffer I. Imidazole was removed by dialysis against this same buffer.

### Structural studies

The secondary structures constitution of trehalase was evaluated by circular dichroism (CD). The CD spectrum was obtained in J-815 Spectropolarimeter (Jasco) using trehalase 1.625 μM in 6.25 mM Tris-HCl pH 8.0, 12.5 mM NaCl. The measurements were done in quartz cuvettes of 0.1 cm light path and recorded as the average of eight consecutive readings between wavelengths of 195 to 260 nm with 0.5 nm intervals. The content of protein secondary structure was obtained using the latest version of CDPro program package [17], and a database of 43 proteins distributed with the package.

Size-exclusion chromatography (SEC), a method that allows estimating the molecular mass of proteins in conditions close to the native state, was applied for inference of the quaternary structure of recombinant Xcc trehalase. For this, trehalase in 25 mM Tris-HCl (pH 8), 50 mM NaCl was applied into a pre-packed Superdex 200 10/300 GL column equilibrated in this same buffer. The assay was performed on a chromatograph ÄKTA Purifier (GE Healthcare Life Sciences) under flow rate of 0.5 mL. min^-1^ of the same buffer, monitoring the absorbance at 280 nm.

### Enzyme activity of recombinant Xcc trehalase

Trehalases catalyze the hydrolysis of a trehalose molecule, resulting in two molecules of glucose. Thus, the glucose formation rate was measured using a colorimetric assay based on glucose oxidase and peroxidase coupled method [18]. It was used trehalose (Sigma) as the substrate and a glucose quantification kit (Glucose Liquiform, Labtest), according to the manufacturer's instructions. All enzymatic assays were done in replicates at 25°C in a 96-well microplate and the absorbance was monitored at 490 nm by an iMark Microplate Reader (Bio-Rad). Reactions were performed with the following final concentrations of trehalose (mM): 0.03125, 0.125, 0.25, 0.5, 1, 2 and 4. For kinetic all reactions were composed of 270 μL solution provided by Glucose Liquiform kit (Labtest), 15 μL of TreA (0.01 mg.mL^-1^) and 15 μL of trehalose solutions at different concentrations. Negative control was done replacing the volume of enzyme solution with buffer I and the correspondent value was subtracted from all measurements.

### Construction of deletion system

A method based on double homologous recombination between the Xcc genomic DNA and suicide vector pNPTS138 (M.R.K.Alley, unpublished data) was used. For this, the 1 kb sequences upstream and downstream the target ORF were separately amplified and cloned into vector pJET 1.2 and then subcloned *in tandem* into pNPTS138 deletion vector [19].

For the upstream region, *Bam*HI and *Eco*RI restriction sites were adapted to the 5' (5' TATGGATCCGGTAAACGACCGGTGTGGCGG) and 3' (5' AGCGAATTCGTCCATCCGGTGCCATTGCAC) ends respectively. For the downstream region, *Hind*III (5'CGACTAAGCTTGTCGGTGACCAGTTCCGGTGT) and *Bam*HI (5' TATAGGATCCGGACGGTGCCGCTGCTGC) sites were adapted. PCR and cloning were performed following the same conditions mentioned previously for trehalase ORF, except for the hybridization temperature, changed to 60.9°C.

### Trehalase ORF deletion

Electrocompetent wild type Xcc was transformed with 250 ng of the constructed deletion vector using a 0.2 cm gap cuvette in a Gene Pulser Xcell (Biorad), using parameters of 2.5 kV pulse, 50 Ω resistance and 50 μF capacitance [20]. The selection and isolation of transformants was done on LB-agar added of kanamycin 40 μg.mL^-1^.

In order to obtain double crossing-over plasmid-free deleted mutants, transformants were grown for 24 h at 30°C and 250 rpm in LB broth without antibiotics. After that, 0.2 μL of the culture were spread over LB agar added of 10% sucrose. The pNPTS138 vector carries a copy of the gene encoding the enzyme levansucrase (SacB of *Bacillus subtilis*), which converts sucrose into a toxic compound to the cells [21]. Thus, only the bacteria which lost the plasmid with no recombination or transferred the target gene to the plasmid by double recombination and then eliminated it (during growth in absence of antibiotic) will grow on medium containing sucrose.

PCR amplification using oligonucleotides that hybridize in the regions of chromosomal DNA adjacent to the 1 kb flanking regions of the target gene (about 50 bp apart, and therefore unable to hybridize to the deletion plasmid) were used to confirm the deletion. Thus, it was possible to differentiate a deleted mutant (XccΔtreA) from a wild type colony according to the sizes of the PCR products (not shown).

#### Enzyme activity of trehalase in cellular lysates

TreA activity in cellular lysates of Xcc, XauC and XccΔtreA was also recorded after bacteria growth in LB, a non-pathogenicity inducing medium, and XAM-M, a pathogenicity inducing (*hrp*-inducing) medium. For this, isolated colonies of Xcc, XauC and XccΔtreA were grown in 30 mL of culture media until OD_600nm_ of 0.4. The cultures were centrifuged at 12,000 x *g* for 15 min, the cell pellet resuspended in 4 mL of buffer II (25 mM Tris-HCl pH 8.0, 50 mM NaCl) and cells lysed by ultrasound pulses in an ice bath. Then, cell lysates were centrifuged at 12,000 x *g* for 15 min at 4°C and the supernatants collected for activity measurements. Reactions were set up in triplicate, all containing 100 μL of each cell lysate, 15 μL of 0.8 M trehalose and 185 μL of solution provided by Glucose Liquiform kit. Negative control had the cell lysate volume replaced by buffer II.

### *In vivo* growth of deletion mutant and wild strain

Both wild type Xcc and XccΔtreA strains were grown in 25 mL of LB until OD_600nm_ of 0.6. The cells were centrifuged 12,000 x *g* for 15 min at 28°C and cell pellets were washed and resuspended in distilled sterile water to OD_600nm_ of 0.4. Each bacterial suspension was used in infiltration of the abaxial surface of four *Citrus aurantifolia* leaves. Ten punctures were made with a needle in each leaf followed by spreading of bacterial suspension. Negative control was distilled sterile water. Development of infections was followed visually along 15 days at 25°C.

## Results and Discussion

### Structural studies

**Fig 1:**
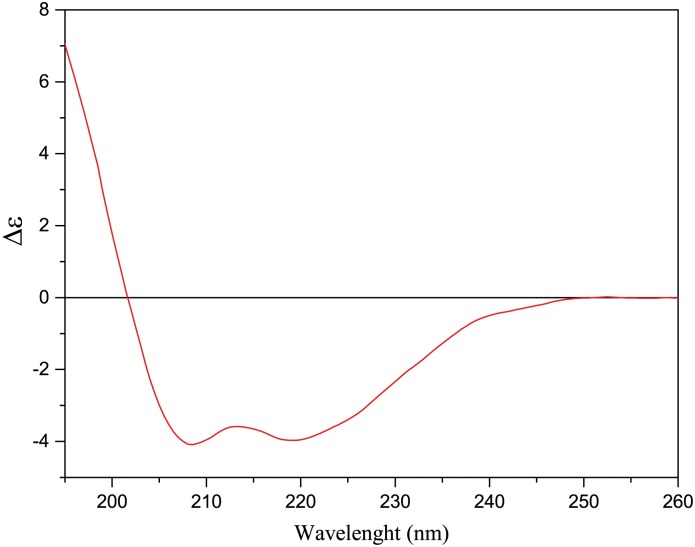
Circular dichroism spectrum of TreA. TreA was analyzed by CD recorded as the mean of eight consecutive readings in the range of 195 to 260 nm. The measurements were performed in a J-815 spectropolarimeter (JASCO) at room temperature using quartz cuvettes of 0.1 cm light path. The values obtained are given in mean residue molar ellipticity (Δε) and the spectrum shows two minimum bands at 219 nm and 208 nm and a negative-positive crossover at 201 nm, typical of structures composed mainly of α-helices.

**Fig 2:**
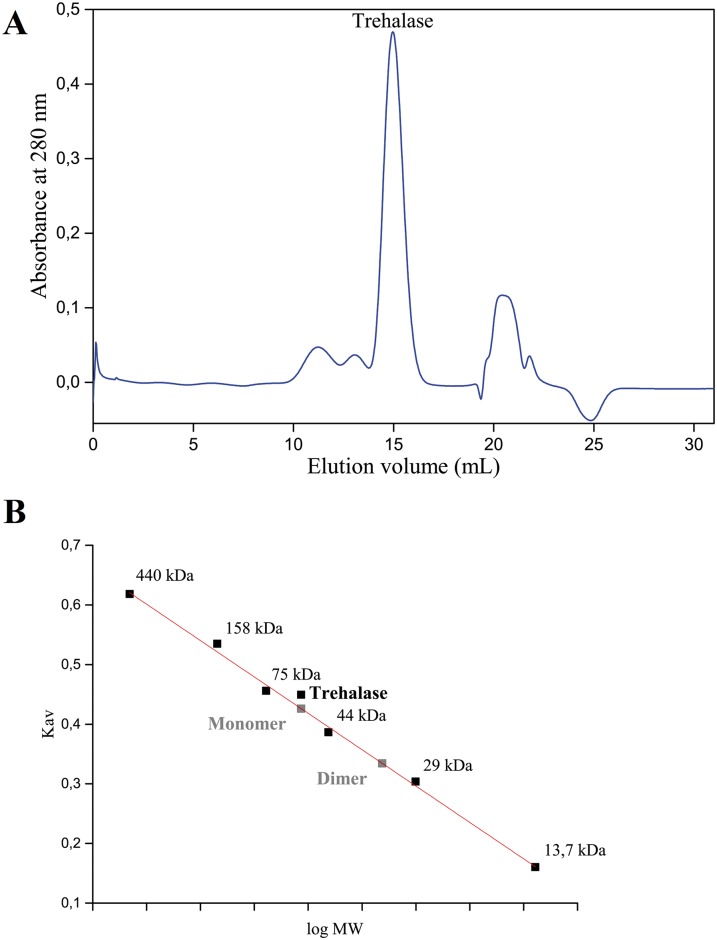
Size-exclusion chromatography of TreA. TreA was subjected to Size-exclusion chromatography (SEC) on a Superdex 200 10/300 GL column (GE Healthcare Life Sciences) at flow rate 0.5 mL/min in 25 mM Tris-HCl pH 8.0, 50 mM NaCl, and monitored by absorbance at 280 nm. (A) Chromatogram indicating TreA major elution peak around 15 mL. (B) Partition coefficients (Kav) of TreA and proteins used in the SEC calibration curve. TreA elution was modeled as a monomer or dimer and the results indicate that TreA must be a monomer. Pearson's linear correlation coefficient was 0.996 for the calibration curve.

Deconvolution of the CD spectrum of TreA (Fig 1) indicated 42.7% of secondary structures in α-helix and 13% in β-sheets, a expected composition given the extensive identity with the trehalase from *E*. *coli*, which has 47% of α-helices and 8% β-sheets [22]. The data obtained from size-exclusion chromatography (SEC, Fig 2) indicated that Xcc TreA must be a monomer in solution.

### Enzyme activity of recombinant TreA

**Fig 3:**
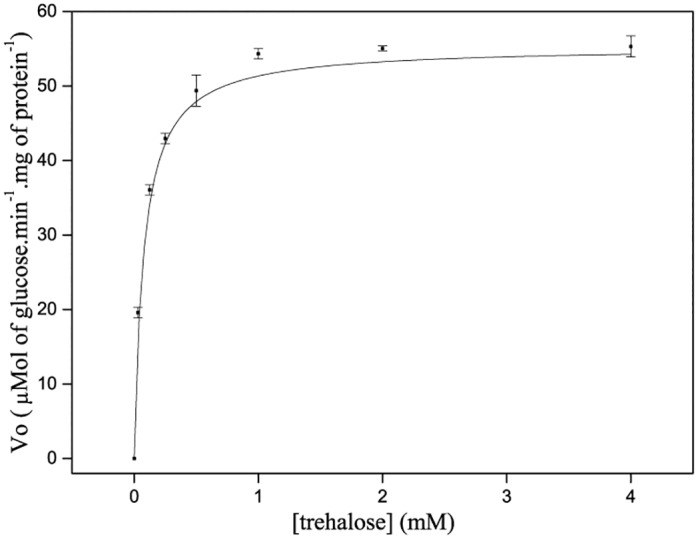
Kinetics studies of recombinant Xcc TreA. Purified protein was used in reactions with different concentrations of substrate trehalose and monitored for glucose production (Glucose Liquiform, Labtest). For each reaction, the initial velocity was correlated with the respective concentrations of trehalose. Data modeled to Michaelis-Menten equation resulted in a maximum speed (V_max_) of 55.308 μmol of glucose.min^-1^.mg protein^-1^. Michaelis dissociation constant (K_M_) for trehalose was 0.077 mM. Error bars represent the mean standard deviation among replicates.

Distinct substrate concentrations were used to calculate recombinant Xcc TreA initial rate (V_0_) of glucose generation (Fig 3). The data were modeled onto Michaelis-Menten equation [23] and K_M_ for the trehalose was 0.077 mM, V_max_ was 55.308 μmol of glucose.min^-1^.mg^-1^ protein. By comparison, *E*. *coli* periplasmic trehalase, the sole periplasmic trehalase with solved structure, recorded K_Ms_ of 0.31, 0.41, 0.8 mM [22, 24, 25]. Different purification methods might explain the activities disparities, including our recombinant Xcc TreA which comes from IMAC. Nevertheless, our expression system produces the predicted mature periplasmic Xcc TreA in the cytosolic compartment, and similar *E*. *coli* periplasmic trehalase constructions showed a K_M_ lowered to 0.16 mM, even though measured from a whole cell extract [24]. These data show similarity in terms of catalytic efficiency, consistent with the predicted secondary (Fig 1) and primary structure homology shared between the enzymes (53.4% identity and 81% similarity).

### Trehalase activity in *Xanthomonas* spp. cellular lysates

Cell lysates from Xcc, XauC and XccΔtreA were cultivated in LB broth and XAM-M, non-pathogenicity and pathogenicity inducing media, respectively, and analyzed for TreA activity. The formation of glucose was used to calculate the initial rate (V_0_) of each of the reactions (Fig 2). Here, initial reaction rates do not have function in characterizing the kinetic behavior of trehalases, but this assay is a way to evaluate the relative amount of the enzyme and in some way the expression of that gene.

**Fig 4:**
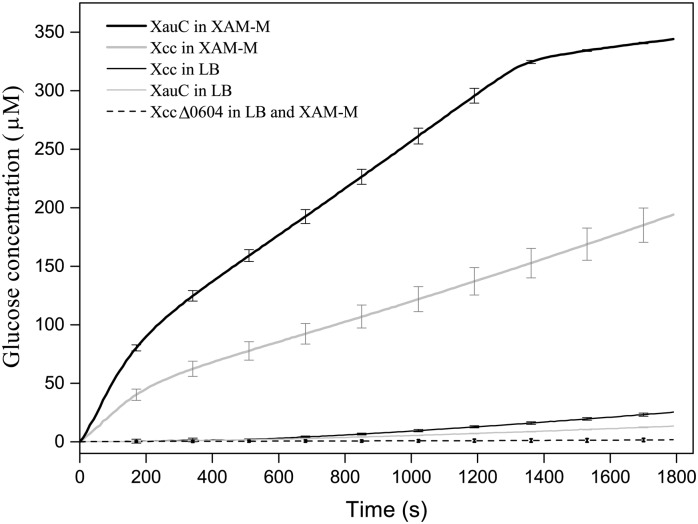
TreA activity in Xcc, XauC and XccΔtreA. The soluble fraction of cell lysates of Xcc, XauC e XACΔtreA grown in LB and XAM-M media were added to trehalose and used in glucose detecting reactions for evaluation of TreA activity. The initial rates of reactions (mM of glucose.L^-1^.s^-1^) using the cell lysates of Xcc in LB, XauC in LB, Xcc in XAM-M and XauC in XAM-M were: 0.0164, 0.007, 0.0952 and 0.1998, respectively. As expected, cell lysates from XccΔtreA did not present TreA activity. Error bars indicate the absolute mean standard deviation among triplicates.

The cell lysate of XccΔtreA showed no TreA activity in both conditions (LB broth and XAM-M), as expected due to gene deletion. Also, cell lysates from Xcc and XauC cells grown in LB medium showed very little TreA activity. Interestingly, in XAM-M *hrp*-inducing medium, XauC presented initial rate of 0.1998 mMol of glucose.L^-1^.s^-1^, about twice the value obtained for Xcc in the same medium. Moreover, mimicking plant environment also triggered an increase of trehalase activity of 10 and 20 times in Xcc and XauC respectively (Fig 4), in comparison with LB medium. This suggests that trehalase should have its expression induced or activity modulated also *in planta*. These findings also corroborate the hypothesis that trehalose could play some role in the infectious process. Concurrently, XauC is less virulent than Xcc [2] although their trehalases sequences are 98% identical.

Considering trehalose as a molecule that increases virulence [26] and its potential role as a protective molecule [27], it is possible that the increased expression of trehalase in XauC relatively to wild type Xcc (Fig 4), and consequent greater trehalose hydrolysis to glucose, could be related to the reduced pathogenicity. However, this speculation is very preliminary and additional studies, are necessary to a better understand the correlation between Xcc virulence and trehalose hydrolysis.

### Deletion of treA increases the virulence of Xcc on *Citrus aurantifolia*

**Fig 5:**
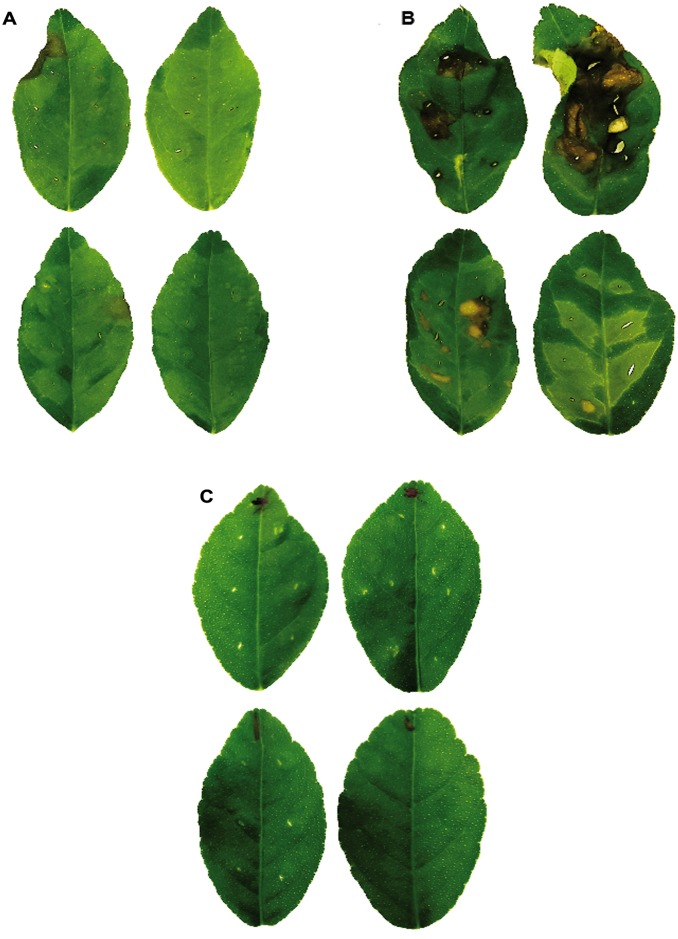
*In vivo* infection with XccΔtreA and Xcc in *Citrus aurantifolia*. Citrus host was artificially infected and used for comparative assessment of pathogenicity of wild type Xcc and XccΔtreA. **A, B and C—**Wild type Xcc, XccΔtreA and negative control, respectively. The photographs were taken five days after infiltration. The leaves infiltrated with XccΔtreA showed higher soaking and necrosis of plant tissue, and intense brownish pustules when compared with the leaves infiltrated with wild type Xcc.

The XccΔtreA strain was tested for pathogenicity in *Citrus aurantifolia*. Infiltrations were made in the abaxial surface of the leaves using equivalent bacterial suspensions of XccΔtreA, wild type Xcc or distilled sterile water as negative control. On the fifth day after the infiltrations, the leaves were detached from the plant for photographic record (Fig 5).

Remarkably, the leaves infiltrated with the XccΔtreA mutant showed much more severe disease symptoms in comparison with the leaves infiltrated with wild type Xcc. The magnitude of the symptoms produced by the XccΔtreA is suggestive that the TreA deleted mutant could be more aggressive than the wild type strain.

Here again, the aggressiveness of XccΔtreA can be understood in the context of trehalose, a molecule not hydrolyzed by TreA in the mutant. Thus, the absence of TreA in XccΔtreA could lead to accumulation of trehalose, since this is not hydrolyzed to glucose. Trehalose is proposed to have protective properties, accumulating under stress situations, such as drying, freezing, osmotic stress and starvation [27]. Conversely, pathogens like *Xanthomonas* could have some colonization advantage in accumulating trehalose, which may explain the greater virulence of XccΔtreA, considering the bacterial stress during infection process.

Although the mechanism of action is not completely understood, the reduction of virulence of microorganisms that do not synthesize trehalose is not uncommon. In *Pseudomonas aeruginosa*, the deletion of two operons related with trehalose biosynthesis led to significant reduction of their growth in *Arabidopsis thaliana* leaves. In addition, when that mutant was co-inoculated with trehalose, the wild type phenotype was restored. Interestingly, this decrease in virulence of the mutant strain was only observed in plant but not in *Caenorhabditis elegans* or *Drosophila melanogaster*, other *P*. *aeruginosa* hosts [28].

In Xcc, the deletion of the trehalose-6-phosphate synthase gene (otsA), that encodes an enzyme that catalyses the first step of the main pathway of trehalose biosynthesis, resulted in the mutant's inability to produce lesions on *Citrus limonia* and *Citrus sinensis* leaves [29], and decreased resistance to salt and oxidative stresses [26]. In *hrp*-inducing XVM2 minimal medium, the mutant strain XccΔotsA produced half of trehalose than Xcc. Moreover, there was no difference in the expression of virulence-related genes (*hrpG*, *hrpX*, *hrpB2* and *gumD*) between wild and mutant strains, suggesting that trehalose does not act neither in the regulation of type III secretion system (T3SS) nor in the production of exopolysaccharides [26].

*Citrus sinensis* leaves infected with XccΔotsA produced less hydrogen peroxide than Xcc. Another interesting fact is that the infiltration of exogenous trehalose into the leaves resulted in the expression of genes for plant oxidative stress response [26], findings that may explain the presence of large necrosis areas in leaves infected by XccΔtreA.

Considering the importance of Xcc trehalase in the infectious process, it is also possible that a plant defense mechanism would increase the expression and/or activity of its own trehalase, a strategy used by *Arabidopsis thaliana* defense against *Plasmodiophora brassicae* infection [30]. Interestingly, either Xcc infection or exogenous trehalose infiltration increased twofold the plant's trehalase activity [26].

## Conclusion

Considering the role of trehalose as a pathogenicity promoter carbohydrate, it is possible that the increased expression of trehalase in XauC relatively to Xcc, consequently greater hydrolysis of trehalose to glucose, could be related to the reduction of its pathogenicity. On the other hand, trehalose maintenance could be related to pathogenicity and explain XccΔtreA infection results. Therefore, it is suggestive that trehalase could be a modulator agent of trehalose availability.

## Supporting Information

S1 Fig Expression and purification of TreA. *E*. *coli* BL21(DE3) were transformed with the expression vector pET28a_treA and subjected to expression, solubility and purification assays on nickel column. (M) protein MW marker (Thermo Scientific). (I) insoluble fraction of lysate. (S) soluble fraction of the lysate. (PC) soluble fraction after passage through the purification column. The protein was eluted by applying a gradient of imidazole concentration (in mM). Arrow indicates the overexpression band of TreA.
(TIF)
