# Correction: treA Codifies for a Trehalase with Involvement in *Xanthomonas citri* subsp. *citri* Pathogenicity

**DOI:** 10.1371/journal.pone.0231024

**Published:** 2020-03-23

**Authors:** André Vessoni Alexandrino, Leandro Seiji Goto, Maria Teresa Marques Novo-Mansur

The affiliation for the first author is incorrect. The correct affiliation is not indicated. André Vessoni Alexandrino is not affiliated with #2 but with: Programa de Pós-Graduação em Biotecnologia—PPGBiotec, Universidade Federal de São Carlos, SP, Brazil.
