# Supplementary figures and images for: treA Codifies for a Trehalase with Involvement in Xanthomonas citri subsp. citri Pathogenicity
Source: PLoS One. 2016 Sep 9;11(9):e0162886. doi: 10.1371/journal.pone.0162886 (PMC5017680; doi:10.1371/journal.pone.0162886)

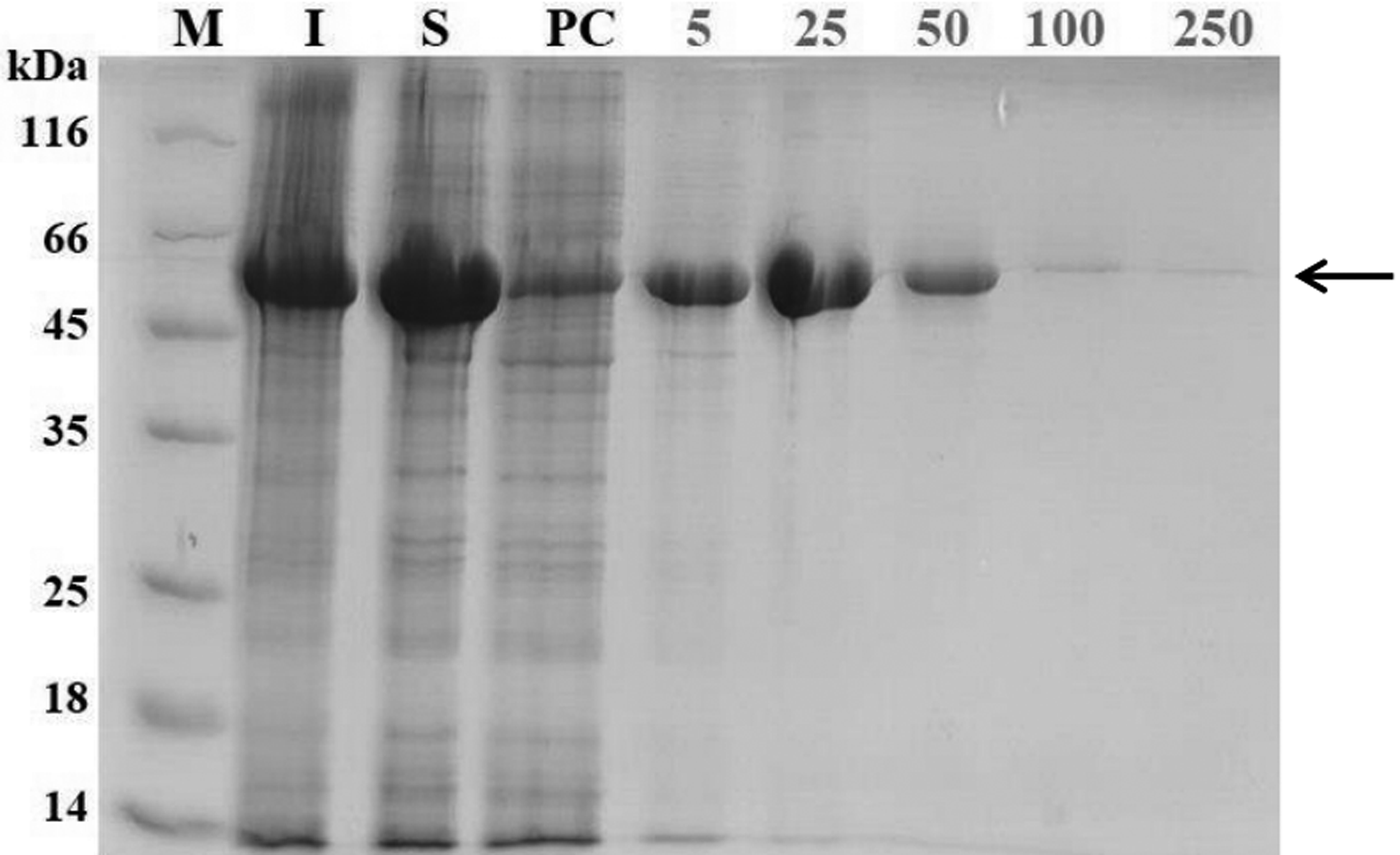

Supplement: S1 Fig — E. coli BL21(DE3) were transformed with the expression vector pET28a_treA and subjected to expression, solubility and purification assays on nickel column. (M) protein MW marker (Thermo Scientific). (I) insoluble fraction of lysate. (S) soluble fraction of the lysate. (PC) soluble fraction after passage through the purification column. The protein was eluted by applying a gradient of imidazole concentration (in mM). Arrow indicates the overexpression band of TreA. (TIF) [file pone.0162886.s001.tif]
